# Parent but Not Peer Attachment Mediates the Relations Between Childhood Poverty and Rural Adolescents’ Internalizing Problem Behaviors

**DOI:** 10.3390/pediatric17050097

**Published:** 2025-09-17

**Authors:** Qingfang Song, Sara S. Whipple, Stacey N. Doan, Rochelle C. Cassells, Gary W. Evans

**Affiliations:** 1 Department of Applied Human Services, Western Kentucky University, Bowling Green, KY 42101, USA; 2 Department of Psychology, Virginia Military Institute, Lexington, VA 24450, USA; whippless@vmi.edu; 3 Department of Psychological Science, Claremont McKenna College, Claremont, CA 91711, USA; stacey.doan@claremontmckenna.edu; 4 Office of Undergraduate Studies, University of Utah, Salt Lake City, UT 84112, USA; rochelle.cassells@utah.edu; 5 Department of Psychology, Cornell University, Ithaca, NY 14850, USA; gwe1@cornell.edu; 6 Department of Human Centered Design, Cornell University, Ithaca, NY 14850, USA

**Keywords:** childhood poverty, parent attachment, peer attachment, externalizing problems, internalizing problems

## Abstract

**Objectives:** The purpose of this study was to examine the prospective, longitudinal relations among childhood poverty and rural adolescents’ internalizing and externalizing problem behaviors, and the mediational roles of adolescent attachment to parents and peers. **Methods:** Participants were from a longitudinal study of rural poverty. Two home visits were conducted, roughly four years apart (Time 1: *N* = 226; *M*age = 13.36, 52.7% male; Time 2: *N* = 215; *M*age = 17.47 years, 51.2% male). Each family’s income-to-needs ratio was assessed at each visit. At Time 2, participants completed questionnaires reporting their attachments to parents and peers, and their externalizing and internalizing symptoms. **Results:** Parent attachment was found to mediate the relationship between Time 1 family income-to-needs ratio and Time 2 internalizing problems. The mediational effects of peer attachment predicting Time 2 internalizing or externalizing symptoms were not significant. **Conclusions:** The long-term impact of childhood poverty on adolescents’ parent attachment and their well-being is discussed.

## 1. Introduction

As of 2021, over 11 million children were identified as living in poverty in the United States [1]. Children under the age of 18 had the highest poverty rate (15.3%) compared to all other age groups, including older adults (10.3%). It is well documented that childhood poverty has adverse impacts on various aspects of children’s physical and mental health (see Refs. [2,3] for reviews). The effect of childhood economic disadvantage appears to extend throughout adolescence and into adulthood [2,4]. Notably, as compared to urban areas, rural areas face elevated risks for both poverty and child problem behaviors [5].

Close relations, particularly the parent–child relationship, have been examined as one important mediator for the effect of childhood poverty on children’s well-being outcomes [6,7,8]. Nevertheless, the extant literature has primarily focused on the role of parent–child relationships in childhood. We know little about the role of other important social relationships during adolescence in their potential contributions to explaining the long-term effect of childhood poverty on rural youth’s psychological adjustment. Adolescence is an important developmental stage whereby peer relations become increasingly important, and peers begin to serve important functions, in part replacing or supplementing the core parent–child relationship [9]. Peers also constitute a significant context for adolescent development [10], as adolescents spend a considerable amount of time with their peers. In this study, we utilized a longitudinal design to explore the mediating roles of parent and peer attachment separately on the relation between childhood poverty and rural adolescents’ psychological adjustment.

### 1.1. Conceptual Framework

In search of mechanisms to explain the effect of childhood poverty on a range of outcomes, Yoshikawa and his colleagues [3] postulated a conceptual framework composed of individual-level, relationship-level, and system-level factors. Relationship-level factors are the interpersonal processes in which children are situated, such as family or peer relationships. They are important drivers of adaptation. Relational factors, however, can be compromised in the context of poverty and related risks [6]. Accordingly, relationship factors may be important mechanisms explaining the link between poverty and psychosocial outcomes in children [7].

As the family stress model specifies, economic hardship can generate stress and consequently strain parenting behaviors [11]. In fact, many studies have demonstrated that relationship processes in the family environment act as potential mediators between family income and child outcomes. Parents in lower-income families were found to engage in harsher and less responsive practices with their children [6,8,12]. For example, parents in impoverished families are more likely to resort to corporal punishment [13], and less likely to support and help children to cope with negative emotions [14]. Nievar and Luster [15] revealed that family income indirectly affected child behavior problems via parenting practices such as positive parent–child interaction and physical punishment. Conger and his colleagues [16] have extensively examined the negative effect of childhood poverty on parenting practices years later. Economic hardship during childhood, as a chronic stressor, continues to increase parenting stress and compromise parents’ capacity to respond to adolescents in need. In turn, the reduced quality in their interactions with parents may mediate or explain the negative link with family poverty and youth’s psychosocial well-being [7].

Furthermore, economic hardship can impact adolescents’ interactions with not only parents but also with peers outside the home environment. Some preliminary evidence has shown that low socioeconomic status or poverty is associated with problematic peer relationships [17,18], possibly because the lack of economic resources may pose difficulty for children in conforming to peer standards and fitting into peer groups [19]. A few recent studies also showed that poverty, particularly material deprivation, is a significant risk factor for experiencing peer victimization at school and thus heightens psychological distress [20] and reduces subjective and psychological well-being [21,22]. While most studies have focused on the effect of poverty on parenting behaviors or peer interactions during childhood, few have tested the potential long-term impact on attachment during adolescence.

### 1.2. Relationship Processes: Family Poverty, Attachment Security, and Youth Behavior

Attachment is conceptualized as the strong affectionate bond established between a child and their primary caregivers to ensure survival [23]. A key factor that determines attachment security is sensitive, responsive parenting [24,25]. Children whose primary caregiver can meet their physical and emotional needs in a prompt and supportive fashion develop a secure attachment with that caregiver, relying on the attachment figure as a secure base from which they can explore the environment and as a safe haven to seek comfort. Secure attachment makes consistent and long-term contributions to children’s psychological well-being, such as competence in emotion regulation, social skills, and self-esteem [23,26].

Notably, while early attachment processes center on the parent–child relationship, across development it often expands to include other significant people [27]. Adolescence is a unique period during which children strive to achieve autonomy from parents and relatedness with peers. Thus, peer interactions and peer relations gain importance during adolescence [28]. In particular, friendships become important contexts for adolescents to develop socioemotional skills and form intimate relationships in a reciprocal manner [29]. Peers in adolescence begin to fulfill many attachment functions, such as providing emotional support in times of distress and serving as safe havens and secure bases [30,31]. In fact, attachment quality with peers is linked with adolescents’ emotional competence [32], life satisfaction [33], and depression in adulthood [34].

While this shift toward peer attachment occurs, secure attachment with parents continues to remain important for adolescent development [32,35]. In terms of trust, communication, and alienation, parent attachment and peer attachment security have been found to make unique contributions to adolescents’ psychosocial well-being [36,37]. Parent and peer attachment are conceptualized to play complementary rather than competing roles in adolescents’ psychosocial adjustment [28,35]

Although the connection between both parent and peer attachment and youth behavior has been established, few studies have examined contextual factors, for example, poverty status, in the development of parent and peer attachment in adolescence. One related study [37] revealed that among adolescents from South Africa, household income shared a unique association with anxious parent attachment, even after controlling for income stability, parental education, and family structure. Additionally, Kenyan adolescents coming from a low SES school or with unemployed mothers were found to have more insecure attachment with parents than their counterparts [38]. The poverty status, as marked by a family income-to-needs ratio severely below the poverty line, predicted the decline of adolescents’ attachment security with parents from age 16 to 18 [39]. Although plausible, existing work for this line of research did not examine the longitudinal effect of poverty specifically on parent attachment during adolescence. Furthermore, there is even less empirical evidence regarding peer attachment in adolescence across different socioeconomic contexts. We know little about the possible roles of adolescents’ parent and peer attachment in mediating the longitudinal impact of childhood poverty on their psychosocial adjustment.

### 1.3. The Current Study

The present study employed a longitudinal design to investigate adolescents’ parent and peer attachments, respectively, as potential mediators for the relationship between childhood poverty and problem behaviors by following a large sample of rural families for over 4 years. Family income-to-needs ratio assessed in middle childhood was used as an index for childhood poverty. In late adolescence, the same participants reported their parent and peer attachment, as well as internalizing and externalizing problem behaviors. Given that economic hardship is associated with less sensitive parenting [7] and more peer problems [20], we expected family income-to-needs ratio in childhood to be associated with adolescents’ attachment security to parents and to peers. Furthermore, we hypothesized that parent and peer attachment security would mediate the relationship between income-to-needs ratio and problem behaviors, such that attachment security would be related to more internalizing and externalizing problem behaviors.

## 2. Materials and Methods

### 2.1. Participants

The study was part of a larger longitudinal project on rural poverty involving multiple time points. Data from all participants that completed assessments during the two time points that fell into the age range were included in the study. The original sample came from rural counties in upstate New York. Low-income families (income-to-needs ≤ 1) were oversampled (53%), while the remaining participants were 2–4 times above the poverty line, the income of most American families. Families were recruited through public schools, New York State Cooperative Extension Programs, and anti-poverty programs. Data for the current study were collected through home visits when participating children were mean ages of 13.36 years (Time 1 [T1]: *N* = 226, 52.7% boys) and 17.47 years (Time 2 [T2]: *N* = 215, 51.2% boys). At Time 2, most participants (68%) were from partnered households, and the rest from divorced/single/widowed households.

### 2.2. Procedures

All data were collected during home visits during which two experimenters worked independently with the participating child and their mother. At each home visit, mothers completed questionnaires to report physical, economic, and psychological stressors, as well as the participating child’s well-being. Meanwhile, the participating children completed a series of tasks to assess their stress and health outcomes. Experimenters went through ethics training on human subjects research and related training on the study protocols prior to conducting home visits. Each participating family received $200 for completing all tasks during each home visit. Tasks used in the current study are described below.

### 2.3. Measures

Income-to-needs ratio. Family income-to-needs ratio was an index of poverty, which was assessed for each child at T1. Income-to-needs was calculated as the total household income divided by the poverty threshold appropriated for the family size as determined by the U.S. federal government. The U.S. federal poverty line is set as an income-to-needs ratio of 1. Family income-to-needs ratio was also assessed at T2. To illustrate the longitudinal influence of poverty on child behavioral problems at T2, we only examined income-to-needs ratio at T1 in the analysis.

Parent and peer attachment. Attachment security was assessed only at T2 through the adapted Inventory of Parent and Peer attachment (IPPA; Ref. [40]). Participating children completed the questionnaire, which taps into three aspects of their relationship with parents and friends, respectively, with a rating scale of 1 (“almost never or never true”) to 5 (“almost always or always true). Composite scores were calculated for three dimensions: degree of mutual trust (e.g., “I trust my friends”), quality of communication (e.g., “I tell my parents about my problems and troubles”), and extent of anger and alienation (e.g., “I get upset a lot more than my parents know about”). A composite score of the three dimensions was used to index the attachment security for each attachment figure (parents, peers). The measurement exhibited good reliability and has been validated in different countries [41]. For the current sample, Cronbach’s alpha ranged from 0.67 to 0.92.

Externalizing and internalizing symptoms. At both T1 and T2, the participating children completed the Youth Self Report Inventory (YSR; Ref. [42]), a widely used, well-validated questionnaire with 112 items indexing youth’s behavioral and emotional problems. Children rated each item on a 3-point scale from 0 (not true) to 2 (very true or often true). The externalizing and internalizing subscales in the present study had good internal consistencies with respective alpha coefficients of 0.87 and 0.90.

## 3. Results

### 3.1. Data Analysis Plan

**Figure 1:**
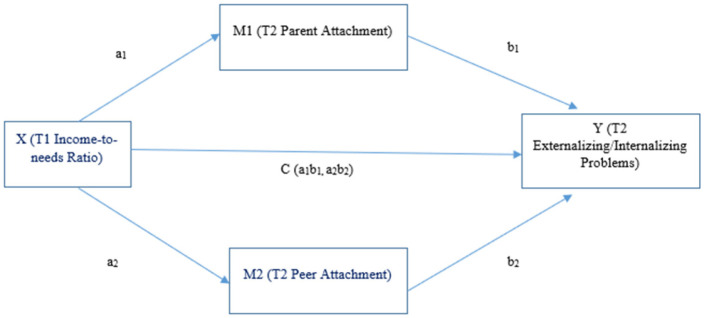
Illustration of parent attachment and peer attachment as mediators.

SPSS 28.0 PROCESS macro was used to test whether parent attachment and peer attachment assessed at T2 would mediate the association of T1 income to needs ratio and T2 internalizing and externalizing symptoms. Using PROCESS macro model 4, two separate mediational models were constructed predicting T2 externalizing and internalizing problems, respectively, with T2 parent attachment and peer attachment as mediating variables, and T1 income-to-needs ratio as the independent variable (See Figure 1). Child demographics (i.e., gender, ethnicity, and age at T1) and internalizing and externalizing problems at T1 were included as covariates. Listwise deletion was used to handle missing values by default.

SPSS PROCESS macro generates a 95% confidence interval based on 5000 bootstrap samples to test the significance of the indirect effect. In accordance with Hayes [43], a variable was deemed to exert a significant indirect effect (mediator) if the confidence interval did not contain zero. The tables list the point estimate and 95% confidence interval for the mediational paths.

### 3.2. Primary Analysis

Table 1 lists the correlations among the study variables. Repeated measure ANOVA analyses showed that adolescents reported more externalizing (*M*_diff_ = 0.02, *SD* = 0.02, *p* = 0.171) and fewer internalizing problems (*M*_diff_ = −0.02, *SD* = 0.02, *p* = 0.314) at T2 as compared to T1, but the differences were not significant. Externalizing and internalizing problems at T1 were significantly associated with those at T2, *r* > 0.40. *p* < 0.001. T1 income-to-needs ratio was correlated with T2 parent attachment but not with peer attachment. Both T2 parent attachment and peer attachment were negatively associated with externalizing and internalizing problems at both time points.

The model 4 in SPSS PROCESS macro revealed significant mediation predicting T2 internalizing problems, as shown in Table 2. After controlling for the effects of age, gender, ethnicity and T1 internalizing problems, the results showed that T1 income to needs ratio had a significant negative effect on T2 parent attachment (a_1_ = 1.90, SE = 0.87, *p* = 0.030). When the mediation variables of T2 parent attachment and peer attachment were added into the model, the effect of T1 income-to-needs ratio on T2 internalizing problems was not significant (c = 0.013, SE = 0.012, *p* = 0.265), and T2 parent attachment still had a significant negative effect on internalizing problems (b_1_ = −0.004, SE = 0.001, *p* < 0.001). Confidence intervals of the indirect effect for T2 parent attachment did not include zero (Boot a_1_b_1_ = −0.04, Boot SE = 0.02, Boot CI: −0.097 to −0.003), indicating a significant mediational effect. On the other hand, T1 income- to-needs did not have a significant effect on T2 peer attachment (a_2_ = 0.53, SE = 0.79, *p* = 0.664). Confidence intervals of the indirect effect of T2 peer attachment included zero (Boot a_2_b_2_ = −0.007, Boot SE = 0.01, Boot CI: −0.034 to 0.016), indicating a nonsignificant mediational effect.

As shown in Table 2, T1 income-to-needs ratio was not correlated with T2 parent (a_1_ = 1.62, SE = 0.89, *p* = 0.0706) or peer attachment (a_2_ = 0.20, SE = 0.80, *p* = 0.801), after controlling for child age, gender, ethnicity, and T1 externalizing problems. The mediational effect of T2 parent attachment (Boot a_1_b_1_ = −0.02, Boot SE = 0.02, Boot CI: −0.058 to 0.001) on peer attachment (Boot a2b2 = −0.00, Boot SE = 0.001, Boot CI: −0.020 to 0.017) predicting T2 externalizing problem was not significant.

## 4. Discussion

A staggering number of studies demonstrate the link between childhood adversity and well-being both concurrently and in later life [7], yet the role of attachment as potential underlying explanation for these linkages has not been examined. This study was among the very few that focused on the long-lasting impact of rural poverty on youth’s psychosocial adjustment. The current study examined the mediating roles of parent and peer attachment in the relationship between family income-to-needs ratio in childhood and externalizing and internalizing problems in rural adolescence.

As hypothesized, parent attachment was found to significantly mediate the relationship between family income-to-needs ratio in childhood and internalizing problems in adolescence. Specifically, children in lower income families during early adolescence were less likely to report secure parental attachment 4 years later. Furthermore, less secure attachment mediated the link between childhood poverty and changes in internalizing problems in children between 13 and 17 years of age. Albeit very small, the effect size of the indirect effect was comparable to other mediational analyses, as a large sample is required to detect a significant mediational effect [44]. The result is also in line with the family stress model which posits that economic disadvantage puts a strain on parenting behaviors [11]. Children whose parents face economic hardship may encounter more insensitive, less responsive parenting behaviors. We also know that less responsive parenting can undermine attachment security [24]. Overall, the mediational results highlight the importance of parent–child relationship processes in transmitting the adverse, long-term impact of childhood poverty among rural families. This finding supports Yoshikawa et al.’s model [5]. In addition, our findings corroborate the proposition that parent attachment continues to function as an important foundation for adolescent adjustment [32], particularly emotional symptoms [36], even though peer relationships become increasingly important with age.

It is noteworthy that the mediating effect was only significant in predicting later internalizing problems but not externalizing problems. Childhood income-to-needs ratio did not make a significant contribution to parent attachment in adolescence, after controlling for prior levels of childhood externalizing problems. This pattern of data suggested child-driven effects, whereby early childhood externalizing problems may have a long-lasting effect on parenting [45]. The nonsignificant result for externalizing problems was also consistent with the finding of a new meta-analysis study which found that children’s externalizing problems did not change as a result of one specific positive parenting intervention, even though this intervention brought positive changes to parenting behavior and attachment [46]. Externalizing problems may be resistant to environmental changes especially for older children. Some other evidence suggested that externalizing problems decline during adolescence [47] and thus the lack of an effect may be due to limited variability.

Unlike parent attachment, peer attachment was not significant in mediating the relationship between childhood poverty and adolescents’ behavior problems, despite previous findings suggesting that socioeconomic status is associated with a wide range of peer problems [17,18,21,22]. One possible explanation may lie in the wide range of life span between T1 (age 13) and T2 (age 17), during which peer relationships may have gone through major changes in childhood and adolescence [48]. Whereas one meta-analysis study [49] demonstrated no significant correlation between age and peer attachment in adolescence, others [50] revealed that adolescents increasingly relied on friends for support and proximity from early to late adolescence, although their affect towards mothers remained stable. Attachment in adolescence is therefore summarized as a combination of stability and changes incurred from changes in relationship quality [51].

Furthermore, peer relationship quality appears to be more conflict-ridden and challenging for rural at-risk adolescents than urban counterparts [52] and the effects of peer network dynamics for rural adolescents need to be assessed. Rural schools may lack resources and be unaccommodating in providing space to cultivate social connection with peers [53,54], which may disrupt the formation of supportive peer attachment and weaken the association between environmental risks and peer attachment in rural adolescents’ lives. Future studies also need to comprehensively examine peer interactions in various contexts, including peer group and school interactions [55], as well as participation in sports and other organized groups [56]. Due to the isolation of rural communities, adolescents are less likely to get involved in organized peer group activities, thus further impeding the formation of secure peer attachment.

Relatedly, it is important to examine various types of peer relationships. In a study of urban Turkish high school students, researchers found that stronger parental attachment was associated with less delinquent behavior among youths, yet attachment to deviant peers was associated with greater delinquent behaviors [57]. This finding suggests that the type of peer influence, not just attachment security alone, is an important consideration. Other work indicates that attachment to best friends was found to have an additional association with adolescents’ depression after controlling for attachment to parents and peers [58]. For late adolescents who are in the transition period to emerging adulthood, engagement and security in romantic relationships may also be uniquely linked to psychosocial adjustment [59]. The benefits of having secure attachment with romantic partners or best friends may be particularly beneficial for those in rural communities, given that rural communities face great barriers to social connections, accessible resources, and interventions [60]. Future studies need to assess and compare attachment of other important relationships to better understand the association between childhood poverty and late adolescents’ psychosocial adjustment.

A few limitations are worth noting. First, the study did not measure attachment to specific other important figures, such as best friends and romantic partners. A more comprehensive investigation of different attachment relationships in adolescents is called for to further elucidate the impact of childhood poverty. Second, parent and peer attachment were only assessed at T2, not T1, therefore making it hard to examine the impact of change of attachment security. Relatedly, the assessment of attachment was based on self-report in the present study which can introduce personal biases. Future studies may adopt observational measures to assess the attachment quality with parents and friends, as well as follow up with adolescents to assess the trajectories or changes in the attachment quality, to further delineate the impact of poverty. Next, the parent attachment measure did not differentiate between attachment to mothers and fathers. Relationship quality with mothers and fathers can have distinct developmental implications in adolescents’ psychosocial adjustment. For example, change in mother-adolescent relationship quality was a significant predictor of self-esteem among both sons and daughters, but the relationship for father-adolescent relationship was only found significant among daughters [61]. Therefore, separate assessments for attachments to mother and to father in future studies may yield more nuanced understanding of the impact of childhood poverty. Furthermore, the longitudinal data spanned from childhood to late adolescence, during which several significant developmental changes in relationships occur over time [60]. Future studies can add more assessments in between the two time points to depict the developmental trajectory of attachment and psychosocial adjustment in relation to childhood poverty.

## 5. Conclusions

To the best of our knowledge, this study was the first to investigate the long-term impact of childhood poverty in rural areas from the attachment perspective. Our findings revealed the mediating effect of parent attachment in the relationship between childhood poverty and changes in rural adolescents’ internalizing problems. Children from families with economic hardship tended to have lower quality of attachment to parents in adolescence which was in turn related to a greater increase in internalizing symptoms over the four-year period. Furthermore, only parental attachment, at least during the maturational period we examined, appears salient, whereas peer attachment did not mediate the poverty–internalizing link. The results can inform the practice of delivering parenting education to families in rural poverty so that parental sensitivity can be enhanced and positive parent–child attachment can be established to break the link between family poverty and child behavioral problems.

## Figures and Tables

**Table 1:** Bivariate correlations among study variables.

	1	2	3	4	5	6	7	8	9	10
1. Gender	-									
2. Ethnicity	−0.10	-								
3. Age T1	−0.07	0.10	-							
4. Externalizing Problems T1	0.01	0.02	0.10	-						
5. Internalizing Problems T1	0.13 ^†^	0.01	0.06	0.69 ***	-					
6. Income-to-needs T1	0.00	0.08	−0.02	−0.26 ***	−0.19 ***	-				
7. Parent Attachment T2	−0.18 **	0.16 *	−0.02	−0.32 ***	−0.33 ***	0.23 **	-			
8. Peer Attachment T2	0.22 **	0.02	−0.12	−0.30 ***	−0.25 ***	0.09	0.42 ***	-		
9. Externalizing Problems T2	0.09	−0.04	0.07	0.59 ***	0.33 ***	−0.19 **	−0.32 ***	−0.29 ***	-	
10. Internalizing Problems T2	0.30 ***	−0.12 ^†^	−0.00	0.39 ***	0.41 ***	−0.03	−0.45 ***	−0.28 ***	0.55 ***	-
*M*	0.49	0.86	13.37	0.41	0.44	2.34	103.64	96.97	0.43	0.41
*SD*	0.50	0.35	1.00	0.27	0.27	1.44	17.82	15.96	0.26	0.27

Note. Gender: 0 = male; 1 = female. Ethnicity. Ethnicity: 0 = non-White; 1 = White. ^†^ *p* < 0.10; * *p* < 0.05; ** *p* < 0.01; *** *p* < 0.001.

**Table 2:** Results of bootstrapping mediation analysis for T2 externalizing and internalizing problems.

Consequent										
Antecedent	Effect IV on M1-PA		Effect IV on M2-PR		Direct Effect on Y		Indirect Effect on Y			
	*β*	*p*	*Β*	*p*	*β*	*p*	Boot B	Boot SE	CI Lower	CI Upper
Y = T2 externalizing, *R*^2^ = 0.42										
X (T1 income-to-needs ratio)	1.51	0.096	0.19	0.817	−0.001	0.599				
M1-PA	-	-	-	-	**−0.002**	**0.029**	−0.02	0.02	−0.058	0.001
M1-PR	-	-	-	-	−0.002	0.172	−0.00	0.01	−0.020	0.016
Gender	−5.99	0.016	7.53	<0.001	0.02	0.567				
Ethnicity	−0.03	0.781	−0.10	0.269	−0.00	0.647				
Age	9.64	0.039	3.01	0.474	−0.07	0.215				
T1 externalizing	−20.40	<0.001	−19.18	<0.001	0.50	<0.001				
Y = T2 internalizing, *R*^2^ = 0.35										
X (T1 income-to-needs ratio)	**1.79**	**0.044**	0.51	0.527	0.01	0.228				
M1-PA	-	-	-	-	**−0.004**	**<0.001**	**−0.04**	**0.02**	**−0.097**	**−0.003**
M1-PR	-	-	-	-	−0.002	0.057	−0.01	0.01	−0.034	0.016
Gender	−4.81	0.057	8.53	<0.001	0.09	0.017				
Ethnicity	−0.05	0.657	−0.12	0.196	−0.00	0.682				
Age	10.08	0.031	3.30	0.436	−0.08	0.172				
T1 internalizing	−20.22	<0.001	−17.47	<0.001	0.31	<0.001				

Note. M1-PA: mediator 1-parent attachment. M2-PR: mediator 2-peer attachment. Gender: 0 = male; 1 = female. Ethnicity. Ethnicity: 0 = non-White; 1 = White. Boot B, Boot SE, and Boot CI for indirect effects were standardized. Paths significant at the 0.05 level are shown in bold.

## Data Availability

Data was available upon the request.

## References

[1] Creamer J., Shrider E.A., Burns K., Chen F. Poverty in the United States: 2021. https://www.census.gov/content/dam/Census/library/publications/2022/demo/p60-277.pdf.

[2] National Academies of Sciences, Engineering, and Medicine (2019). A Roadmap to Reducing Child Poverty.

[3] Yoshikawa H., Aber J.L., Beardslee W.R. (2012). The effects of poverty on the mental, emotional, and behavioral health of children and youth: Implications for prevention. Am. Psychol..

[4] Evans G.W. (2016). Childhood poverty and adult psychological well-being. Proc. Natl. Acad. Sci. USA.

[5] Robinson L.R., Holbrook J.R., Bitsko R.H., Hartwig S.A., Kaminski J.W., Ghandour R.M., Peacock G., Heggs A., Boyle C.A. (2017). Differences in health care, family, and community factors associated with mental, behavioral, and developmental disorders among children aged 2–8 years in rural and urban areas—United States, 2011–2012. Surveill. Summ..

[6] Mazza J.R., Pingault J.-B., Booij L., Boivin M., Tremblay R., Lambert J., Zunzunegui M.V., Côté S. (2016). Poverty and behavior problems during early childhood: The mediating role of maternal depression symptoms and parenting. Int. J. Behav. Dev..

[7] La Placa V., Corlyon J. (2016). Unpacking the Relationship between Parenting and Poverty: Theory, Evidence and Policy. Soc. Policy Soc..

[8] Wadsworth M.E., Evans G.W., Grant K., Carter J.S., Duffy S., Cicchetti D. (2016). Poverty and the development of psychopathology. Developmental Psychopathology.

[9] Hazan C., Zeifman D., Bartholomew K., Perlman D. (1994). Sex and the psychological tether. Attachment Processes in Adulthood.

[10] Steinberg L., Morris A.S. (2001). Adolescent development. Annu. Rev. Psychol..

[11] Conger R.D., Conger K.J. (2002). Resilience in midwestern families: Selected findings from the first decade of a prospective, longitudinal study. J. Marriage Fam..

[12] Pereira M., Negrão M., Soares I., Mesman J. (2015). Predicting harsh discipline in at-risk mothers: The moderating effect of socioeconomic deprivation severity. J. Child Fam. Stud..

[13] Cuartas J., Grogan-Kaylor A., Ma J., Castillo B. (2019). Civil conflict, domestic violence, and poverty as predictors of corporal punishment in Colombia. Child Abus. Negl..

[14] Highlander A., Zachary C., Jenkins K., Loiselle R., McCall M., Youngstrom J., McKee L.G., Forehand R., Jones D.J. (2021). Clinical Presentation and Treatment of Early-Onset Behavior Disorders: The Role of Parent Emotion Regulation, Emotion Socialization, and Family Income. Behav. Modif..

[15] Nievar M.A., Luster T. (2006). Developmental processes in African American families: An application of McLoyd’s theoretical model. J. Marriage Fam..

[16] Conger R.D., Elder G.H. (1994). Families in Trouble Times: Adapting to Change in Rural America.

[17] Olsson E. (2007). The economic side of social relations: Household poverty, adolescents’ own resources and peer relations. Eur. Sociol. Rev..

[18] Schmiedeberg C., Schumann N. (2019). Poverty and adverse peer relationships among children in Germany: A longitudinal study. Child Indic. Res..

[19] Bukowski W.M., Dirks M., Infantino E., Delay D. (2021). Principles for studying contextual variations in peer experiences: Rules for peer radicals. J. Appl. Dev. Psychol..

[20] Chen J.K., Wang Z., Wong H., Tang V. (2021). Child deprivation as a mediator of the relationship between family poverty, bullying victimization, and psychological distress. Child Indic. Res..

[21] Chen J.-K., Wang S.-C., Chen Y.-W. (2023). Social relationships as mediators of material deprivation, school bullying victimization, and subjective well-being among children across 25 countries: A global and cross-national perspective. Appl. Res. Qual. Life.

[22] Jiang S. (2020). Psychological well-being and distress in adolescents: An investigation into associations with poverty, peer victimization, and self-esteem. Child. Youth Serv. Rev..

[23] Bowlby J. (1969). Attachment and Loss. Vol. 1: Attachment.

[24] Atkinson L., Goldberg S., Vaishali R., Pederson D., Benoit D., Moran G., Poulton L., Myhal N., Zwiers M., Gleason K. (2005). On the relation between maternal state of mind and sensitivity in the prediction of infant attachment security. Dev. Psychol..

[25] De Wolff M.S., Van Ijzendoorn M.H. (1997). Sensitivity and attachment: A meta-analysis on parental antecedents of infant attachment. Child Dev..

[26] Brumariu L.E. (2015). Parent-child attachment and emotion regulation. New Dir. Child Adolesc. Dev..

[27] Zimmermann P. (1999). Structure and functions of internal working models of attachment and their role in emotion regulation. Attach. Hum. Dev..

[28] Hazan C., Campa M. (2013). Human Bonding: The Science of Affectional Ties.

[29] Furman W. (2002). The emerging field of adolescent romantic relationships. Curr. Dir. Psychol. Sci..

[30] Hazan C., Zeifman D., Cassidy J., Shaver P.R. (1999). Pair bonds as attachments: Evaluating the evidence. Handbook of Attachment: Theory, Research, and Clinical Application.

[31] Rosenthal N.L., Kobak R. (2010). Assessing adolescents’ attachment hierarchies: Differences across developmental periods and associations with individual adaptation. J. Res. Adolesc..

[32] Laible D. (2007). Attachment with parents and peers in late adolescence: Links with emotional competence and social behavior. Personal. Individ. Differ..

[33] Aziz M., Khan W., Amin F., Khan M.F. (2021). Influence of Parenting Styles and Peer Attachment on Life Satisfaction Among Adolescents: Mediating Role of Self-Esteem. Fam. J..

[34] Cook S.H., Heinzer J.E., Miller A.L., Zimmerman M.A. (2016). Transitions in friendship attachment during adolescence are associated with developmental trajectories of depression through adulthood. J. Adolesc. Health.

[35] Allen J.P., Tan J., Cassidy J., Shaver P. (2016). The multiple facts of attachment in adolescence. Handbook of Attachment.

[36] Oldfield J., Humphrey N., Hebron J. (2016). The role of parental and peer attachment relationships and school connectedness in predicting adolescent mental health outcomes. Child Adolesc. Ment. Health.

[37] Rawatlal N., Pillay B.J., Kliewer W. (2015). Socioeconomic status, family-related variables, and caregiver-adolescent attachment. S. Afr. J. Psychol..

[38] Wambua G.N., Obondo A., Bifulco A., Kumar M. (2018). The role of attachment relationship in adolescents’ problem behavior development: A cross-sectional study of Kenyan adolescents in Nairobi city. Child Adolesc. Psychiatry Ment. Health.

[39] Allen J.P., McElhaney K.B., Kuperminc G.P., Jodl K.M. (2004). Stability and change in attachment security across adolescence. Child Dev..

[40] Armsden G.C., Greenberg M.T. (1987). The inventory of parent and peer attachment: Individual differences and their relationship to psychological well-being in adolescence. J. Youth Adolesc..

[41] Pace C.S., Martini P.S., Zavattini G.C. (2011). The factor structure of the Inventory of Parent and Peer Attachment (IPPA): A survey of Italian adolescents. Personal. Individ. Differ..

[42] Achenbach T. (1991). Child Behavior Checklist.

[43] Hayes A.F. (2013). Mediation, Moderation, and Conditional Process Analysis.

[44] Pan H., Liu S., Miao D., Yuan Y. (2018). Sample size determination of mediation analysis of longitudinal data. BMC Med. Res. Methodol..

[45] Yan N., Ansari A., Peng P. (2021). Reconsidering the relation between parental functioning and child externalizing behaviors: A meta-analysis on child-driven effects. J. Fam. Psychol..

[46] Van IJzendoorn M., Schuengel C., Wang Q., Bakermans-Kranenburg M. (2023). Improving parenting, child attachment, and externalizing behaviors: Meta-analysis of the first 25 randomized controlled trials on the effects of Video-feedback Intervention to promote Positive Parenting and Sensitive Discipline. Dev. Psychopathol..

[47] Bongers I.L., Koot H.M., Der Ende J.V., Verhulst F.C. (2024). Developmental trajectories of externalizing behaviors in childhood and adolescence. Child Dev..

[48] Sholte R.H., Van Aken M.A. (2020). Peer relations in adolescence. Handbook of Adolescent Development.

[49] Gorrese A., Ruggieri R. (2012). Peer attachment: A meta-analytic review of gender and age differences and associations with parent attachment. J. Youth Adolesc..

[50] Nickerson A., Nagle R.J., Dannerbeck A., Casas F., Sadurni M., Coenders G. (2004). The influence of parent and peer attachments on life satisfaction in middle childhood and early adolescence. Quality-of-Life Research on Children and Adolescents.

[51] Allen J.P., Grande L., Tan J., Loeb E. (2018). Parent and peer predictors of change in attachment security from adolescence to adulthood. Child Dev..

[52] Dupéré V., Goulet M., Archambault I., Dion E., Leventhal T., Crosnoe R. (2019). Circumstances preceding dropout among rural high school students: A comparison with urban peers. J. Res. Rural Educ..

[53] Gristy C. (2012). The central importance of peer relationships for student engagement and well being in a rural secondary school. Pastor. Care Educ..

[54] Sherman J., Sage R. (2011). Sending off all your good treasures: Rural Schools, brain-drain, and community survival in the wake of economic collapse. J. Res. Rural Educ..

[55] Millings A., Buck R., Montgomery A., Spears M., Stallard P. (2012). School connectedness, peer attachment, and self-esteem as predictors of adolescent depression. J. Adolesc..

[56] McGee R., Williams S., Howden-Chapman P., Martin J., Kawachi I. (2006). Participation in clubs and groups from childhood to adolescence and its effects on attachment and self-esteem. J. Adolesc..

[57] Yuksek D.A., Solakoglu O. (2016). The relative influence of parental attachment, peer attachment, school attachment, and school alienation on delinquency among high school students in Turkey. Deviant Behav..

[58] Wilkinson R.B. (2010). Best friend attachment versus peer attachment in the prediction of adolescent psychological adjustment. J. Adolesc..

[59] Bar A.B., Sutton T.E., Simons L.G., Wickrama K.A.S. (2016). Romantic relationship transitions and changes in health among rural, White young adults. J. Fam. Psychol..

[60] Bellamy G.R., Bolin J.N., Gamm L.D. (2011). Rural health people 2010, 2020, and beyond: The need goes on. Fam. Community Health.

[61] Keiser R., Helmerhorst K.O.W., Gelderen L.v.R. (2019). Perceived quality of the mother-adolescent and father-adolescent attachment relationship and adolescents’ self-esteem. J. Youth Adolesc..

